# Supplementary material for: End Sequence Analysis Toolkit (ESAT) expands the extractable information from single-cell RNA-seq data
Source: Genome Res. 2016 Oct;26(10):1397–410. doi: 10.1101/gr.207902.116 (PMC5052061; doi:10.1101/gr.207902.116)
Supplement: Supplemental Material [file supp_gr.207902.116_Supp_Materials.tar.gz › ESAT-esat_v0.1/doc/html/interfacebroad_1_1core_1_1annotation_1_1_feature.html]

ESAT: broad.core.annotation.Feature Interface Reference


ESAT


- Main Page
- Related Pages
- Packages
- Classes
- Files

- Class List
- Class Index
- Class Hierarchy
- Class Members

All Classes Namespaces Files Functions Variables Enumerator Pages

- broad
- core
- annotation
- Feature

Public Member Functions |
List of all members

broad.core.annotation.Feature Interface Reference

Inheritance diagram for broad.core.annotation.Feature:

[legend]

|  |  |
| --- | --- |
| Public Member Functions | |
| String | getName () |
|  | |
| double | getScore () |
|  | |

## Member Function Documentation

|  |  |  |  |  |
| --- | --- | --- | --- | --- |
| String broad.core.annotation.Feature.getName | ( |  | ) |  |

Here is the caller graph for this function:

|  |  |  |  |  |
| --- | --- | --- | --- | --- |
| double broad.core.annotation.Feature.getScore | ( |  | ) |  |

Implemented in broad.pda.datastructures.Alignments.

---

The documentation for this interface was generated from the following file:

- java/broad/core/annotation/Feature.java


---

Generated on Fri Aug 15 2014 15:10:51 for ESAT by  

 1.8.7
